# Polyester dendrimers: a versatile nanocarrier for target specific drug delivery

**DOI:** 10.1186/1471-2334-12-S1-P5

**Published:** 2012-05-04

**Authors:** A Aravind, A Deepu, K Santhosh Kumar

**Affiliations:** 1 Chemical Biology Lab, Rajiv Gandhi Centre for Biotechnology, Thiruvananthapuram, India

## Background

Improving the therapeutic index of drugs is a major impetus for innovation in many therapeutic areas such as HIV, cancer, inflammatory and other infectious diseases. Polyester based dendrimers constitute a very attractive class of materials because they are less toxic, biodegradable and biocompatible. Our aim is to develop a novel ester based versatile dendritic nanocarrier that could specifically deliver drugs against HIV, cancer and other infectious diseases.

## Methods

The monomer molecule was chemically synthesized and characterized for the development of nanocarrier. The poly ester based dendrimeric nanocarrier was synthesized by using solid phase organic synthesis. The multifunctional nanocarrier was characterized by UV, IR, NMR, MALDI-TOF, optical scattering and TEM. In-vitro toxicity studies, bioavailibity and biocompatibity studies were carried out using specific receptor over expressing cell lines.

## Results

Novel core-shell type polymer with surface specific amino functional groups was used for the nanocarrier synthesis. The solid phase Michael addition reaction was carried out using double ester acrylate monomer with amino group of the linker. The dendrimeric nanocarrier was allowed to develop up to the 3^rd^ generation. The drug molecule was attached to the surface of the carrier using enzyme cleavable tetrapeptide spacer. The targeting ligands were attached on the surface of the nanocarrier for in-vitro studies.

## Conclusion

This novel dendrimeric nanocarrier with multiple functional sites has tremendous potential as drug delivery vehicle. It could revolutionize the field of chemotherapy and the delivery of toxic, insoluble anti HIV, anticancer and peptide based drugs of short half life with reduced cytotoxicity.

